# Optimal time of delivery to reduce the risk of infant mortality in small and normally grown fetuses: A national cohort study in Korea

**DOI:** 10.1371/journal.pone.0209308

**Published:** 2018-12-14

**Authors:** Hyun Sun Ko, Jeong Ha Wie, Sae Kyung Choi, In Yang Park, Yong-Gyu Park, Jong Chul Shin

**Affiliations:** 1 Department of Obstetrics and Gynecology, College of Medicine, The Catholic University of Korea, Seoul, Republic of Korea; 2 Department of Biostatistics, College of Medicine, The Catholic University of Korea, Seoul, Republic of Korea; University of Liverpool, UNITED KINGDOM

## Abstract

**Purpose:**

To examine the competing risks of stillbirth versus infant death and to evaluate the optimal time of delivery in the population of small for gestational age (SGA) and non-SGA late preterm and term fetuses.

**Methods:**

This was a retrospective national cohort study of all singleton births between 34 0/7 and 42 6/7 weeks of gestation using the Korean vital statistics (n = 2,106,159). We compared the risk of infant mortality with a composite of fetal–infant mortality risk that would occur after expectant management for one additional week and evaluated the optimal time of delivery, in SGA and non-SGA pregnancies.

**Results:**

In the total population, the risk of expectant management became significantly higher than the risk of delivery, at 39 weeks and beyond, similar with non-SGA group. In the SGA group, the risk of stillbirth was significantly greater at all GAs than for non-SGA pregnancies, and the risk of infant death was significantly increased until 38 weeks (25.8 per 10,000 live births, 95% CI 20.11–32.47), and the risk of stillbirth was significantly increased at 41 weeks (11.65 per 10,000 ongoing pregnancies, 95% CI 6.95–18.09), compared to 39 weeks (12 per 10,000 live births, 95% CI 8.98–15.64 and 5.12 per 10,000 ongoing pregnancies, 95% CI 3.84–6.66, respectively).

**Conclusion:**

In Korean women, delivery between 39 and 41 weeks minimizes fetal/infant mortality, in non-SGA pregnancies. In uncomplicated SGA pregnancies, delivery between 39 and 40 weeks can be considered to decrease risk of infant death and stillbirths.

## Introduction

It is important to balance the risks and benefits of delivery when determining the optimal time of delivery, especially when an elective cesarean delivery or induction of labor is scheduled, during term gestation. Stillbirth and infant death are two adverse perinatal outcomes that demonstrate substantial disparities according to race/ethnicity, maternal age, pre-pregnancy body mass index, birth weight, and so on [1]. It is well known that black women are at greatest risk of fetal or infant death [2–4]. It has been reported that Hispanic and Asian women have rates of stillbirth similar to those seen in non-Hispanic white women with lower rates of infant death [2,5]. The prevalence of stillbirth tends to increase with gestational age (GA), although the magnitude varies greatly, with the highest risk seen in black women and the lowest risk seen in white women [2,3,6]. Due to the significant neonatal mortality and morbidities in late preterm (defined as gestational age from 34 weeks and 0 days to 36 weeks and 6 days) and early term birth (defined as as gestational age from 37 weeks and 0 days to 38 weeks and 6 days), an American College of Obstetricians and Gynecologists (ACOG) Committee Opinion stated that women should not be delivered without medical indication prior to 39 weeks’ gestation [7].

The ACOG suggested delivery at 38 0/7–39 6/7 weeks of gestation in cases of isolated fetal growth restriction and a delivery at 34 0/7–37 6/7 weeks of gestation in cases of fetal growth restriction with additional risk factors for adverse outcome (e.g., oligohydramnios, abnormal umbilical artery Doppler velocimetry results, maternal risk factors, or co-morbidities) [8]. However, existing data regarding the timing of delivery in cases of fetal growth restriction is still limited, especially in an Asian population [9]. As neonatal intensive care improves, a larger proportion of children with complications resulting from GA or intrapartum events may be surviving beyond the neonatal period, contributing to a decrease in neonatal mortality rates over time [4]. Also, recent data demonstrated that term infants who die within the first year of life are more likely to do so within the postneonatal period (aged 29–365 days of life) than in the neonatal period [10].

In this study, we investigated the risks of stillbirth and infant death after birth in Korean women during late preterm and term pregnancies. In addition, this study attempted to evaluate the optimal time of delivery, by examining the competing risks of stillbirth versus infant death in small for gestational age (SGA) and non-SGA fetuses.

## Methods

De-identified Korean Vital Statistics Birth Certificate, Vital Statistics Death Certificate, and Vital Statistics Fetal Death File data [11] on a total of 2,297,876 newborns in singleton pregnancies were obtained from Statistics Korea. Korean Vital Statistics is a nationwide database which was developed to understand birth, death, marriage, and divorce, i.e. the basic causes of change in the population size and population structure of Korea. Data from Korean Vital Statistics is released monthly and annually via a press release, on the website (http://kosis.kr), and in online publications, such as the ‘Annual Report on the Vital Statistics.’

Raw data on all fetuses delivered from 2010 to 2014 (470,171 in 2010, 471,265 in 2011, 484,550 in 2012, 436,455 in 2013, and 435,435 in 2014) were analyzed. Our results were based on 2,106,159 births after exclusions. Pregnancy dating was determined using the best obstetric estimate as opposed to the last menstrual period. However, if the estimated age based on the last menstrual period was significantly different from that estimated by ultrasound, GA by early ultrasound was used for this purpose and there was no allowance for GA correction following birth. GA is referred to by interval, in completed weeks; so, for example, a GA of 40 weeks means 40 weeks plus 0–6 days. BW was measured to the nearest 10 g. This study used anonymous registry data and we obtained approval from the institutional review boards of Catholic University of Korea (KC17ZESI0171).

### Inclusion and exclusion criteria

The study population comprised all singleton deliveries to Korean mothers between 34 and 42 6/7 weeks of gestation. We excluded fetal or infant deaths caused by congenital abnormalities in the death registry. Newborns with unknown body weight or GA (n = 4,624), GA <34 weeks or > 42 weeks (n = 35,149), multiple pregnancies (n = 74,938), non-Korean nationality (n = 99,931), large for gestational age (> 95 percentile, n = 120,095), extreme maternal age (< 15 or > 45 years old, n = 2,814) were excluded. Some of the exclusions (n = 30,492) were duplicated.

### Small for gestational age groups

We defined SGA as birth weight below the 10th percentile for a particular gestational age. Therefore, we stratified our analysis into an SGA group (birth weight by gestational age below the 10th percentile) and a non-SGA group (birth weight by gestational age between the 10th and 95th percentiles, according to Korean reference curves for birth weight by gestational age, sex, and plurality of newborns in Korea [12].

### Outcome measures

Stillbirth was defined as intrauterine fetal death occurring after 20 weeks’ GA and before the start of delivery or those occurring during labor. Infant death was defined as death occurring within the first year of life. Infant mortality rate at each GA was calculated as the number of infant death per 1,000 live births at that same GA and fetal mortality rate at each GA was calculated as the number of fetal deaths per 1,000 live births and fetal deaths at that same GA. The risk of stillbirth at a given GA was calculated as the number of stillbirths (whether antepartum or intrapartum) at that GA per 10,000 ongoing pregnancies, minus half of the births in the given week. This correction factor (subtracting half of the deliveries during the week of investigation from the denominator of total ongoing pregnancies) was described by Smith as a way to correct for the censoring of pregnancies that are delivered during the week, assuming they occurred on average half-way through the week [5, 13]. The mortality risk of delivery during a given week was defined as the mortality rate among infants born during that gestational week. The mortality risk of 1 week of expectant management was defined as the risk of stillbirth over that week plus the mortality risk experienced by infants born in the subsequent week of gestation. As described in detail previously [5], the composite risk of expectant management for 1 week represents the sum of the probability of stillbirth during a given week of gestation plus the probability of infant death when birth occurs in the subsequent week. This composite risk of expectant management beyond each given week of gestation was then compared with the risk of infant death for children born in the given week of gestation. The “number needed to deliver” was calculated as an analogous measure as the “number needed to treat” by taking the reciprocal of the absolute difference in risk between delivery and expectant management.

### Statistical analysis

Statistical calculations were performed with Excel and SAS version 9.3 (SAS Institute Inc., Cary, NC, USA), including proportions, relative risks, and 95% confidence intervals (CIs). Chi square tests were performed to compare proportions of independent variables and t-test was performed to compare means. Poisson regression analysis was performed to compare risk of stillbirth, infant death, and expectant management by gestational age. Statistical significance was reached with a P value of <.05 or if the 95% CIs did not overlap. We assumed that the binomial probability distributions of both mortality risks approximated the normal distribution and derived the CI of the composite risk using the sum of the variances plus twice the covariance of the estimates of infant death and stillbirth.

## Results

**Fig 1:**
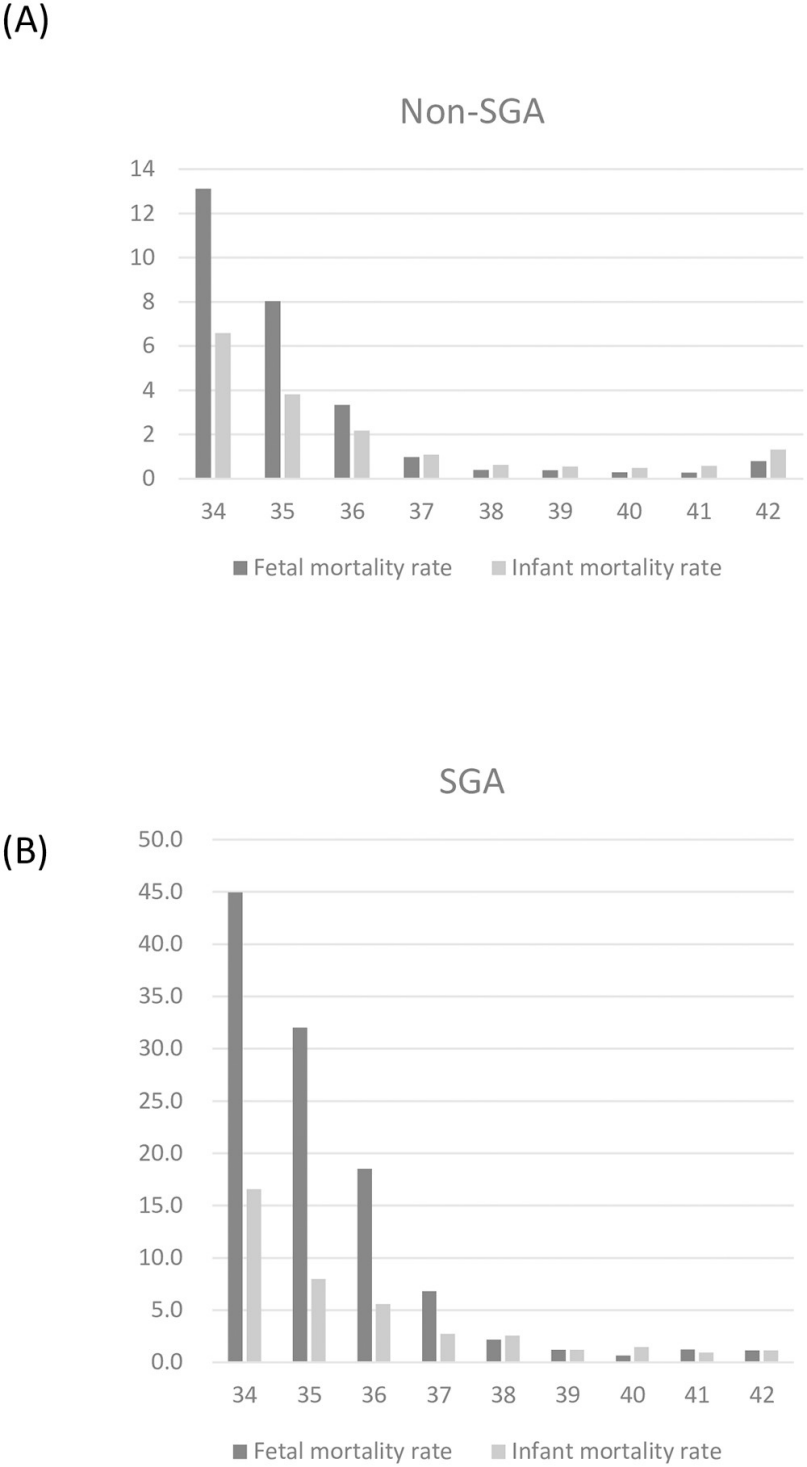
Fetal mortality rates and infant mortality rates at each gestational week. (A) non-small for gestational age (SGA) group (B) SGA group.

Our analysis included 2,106,159 deliveries, of which 142,091 (6.75%) were SGA and 1,964,068 (93.25%) were non-SGA. In our cohort, there were 1,589 total stillbirths, of which 406 were in the SGA group and 1,183 were in the non-SGA group. There were 1638 total infant deaths, of which 275 were in the SGA group and 1,363 were in the non-SGA group. Maternal and infant baseline characteristics of the study cohort are shown in Table 1. Infant mortality rate and fetal mortality rate at each GA were presented in Fig 1.

**Table 1:** Demographic characteristics of women with singleton gestations between 34 and 42 weeks of gestation in Korea between 2010 and 2014.

	Stillbirth (n = 1,589)			Infant Death (n = 1638)			Alive (n = 2,106,159)		
	Non-SGA n = 1183	SGA n = 406	*p*	Non-SGA n = 1363	SGA n = 275	*p*	Non-SGA n = 1964068	SGA n = 142091	*p*
**Maternal age (y)**	31.5±4.4	31.8±5	0.165	31±4.9	30.9±5.3	0.642	31.3±3.9	31±4.2	<.001
<35	917(77.51)	288(70.94)	0.008	1046(76.74)	214(77.82)	0.699	1588449(80.88)	116359(81.89)	<.001
≥35	266(22.49)	118(29.06)		317(23.26)	61(22.18)		375619(19.12)	25732(18.11)	
**Gestational age (wk)**	37.4±2	36.9±2	<.001	38.2±1.7	38.1±1.9	0.737	38.8±1.2	39±1.4	<.001
**More than 12 y of education**	437(96.9)	175(94.09)	0.097	1013(95.03)	184(95.83)	0.633	1939361(98.92)	139311(98.24)	<.001
**Birthweight (g)**	2953.5±465.4	2038.5±558.8	<.001	3118±389.8	2246.8±569.4	<.001	3302.8±365.6	2593.4±271.3	<.001
**Male sex**	587(49.62)	231(56.9)	0.011	766(56.2)	157(57.09)	0.786	1008728(51.36)	73396(51.65)	0.032

Data are mean±standard deviation or n (%) unless otherwise specified. SGA, small for gestational age, n, number, wk, week

### Total cohort

**Fig 2:**
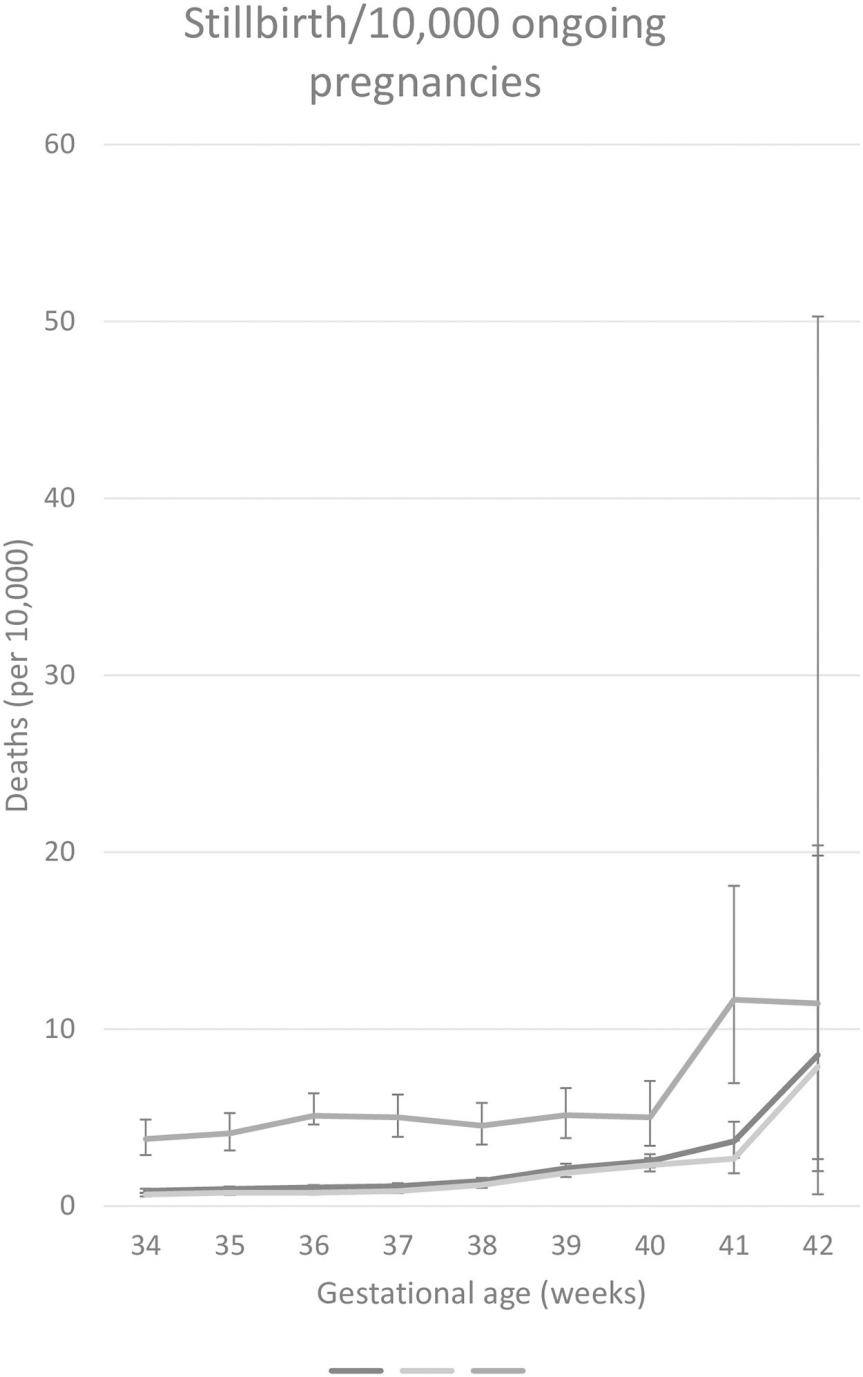
Stillbirth rate. Stillbirth rates in the total population and the non-small for gestational age(SGA) group shows an increasing pattern at 42 weeks. The stillbirth rate in the SGA group is higher than that in the other groups, at all gestational ages, and it shows an increasing pattern from 41 weeks onwards.

**Fig 3:**
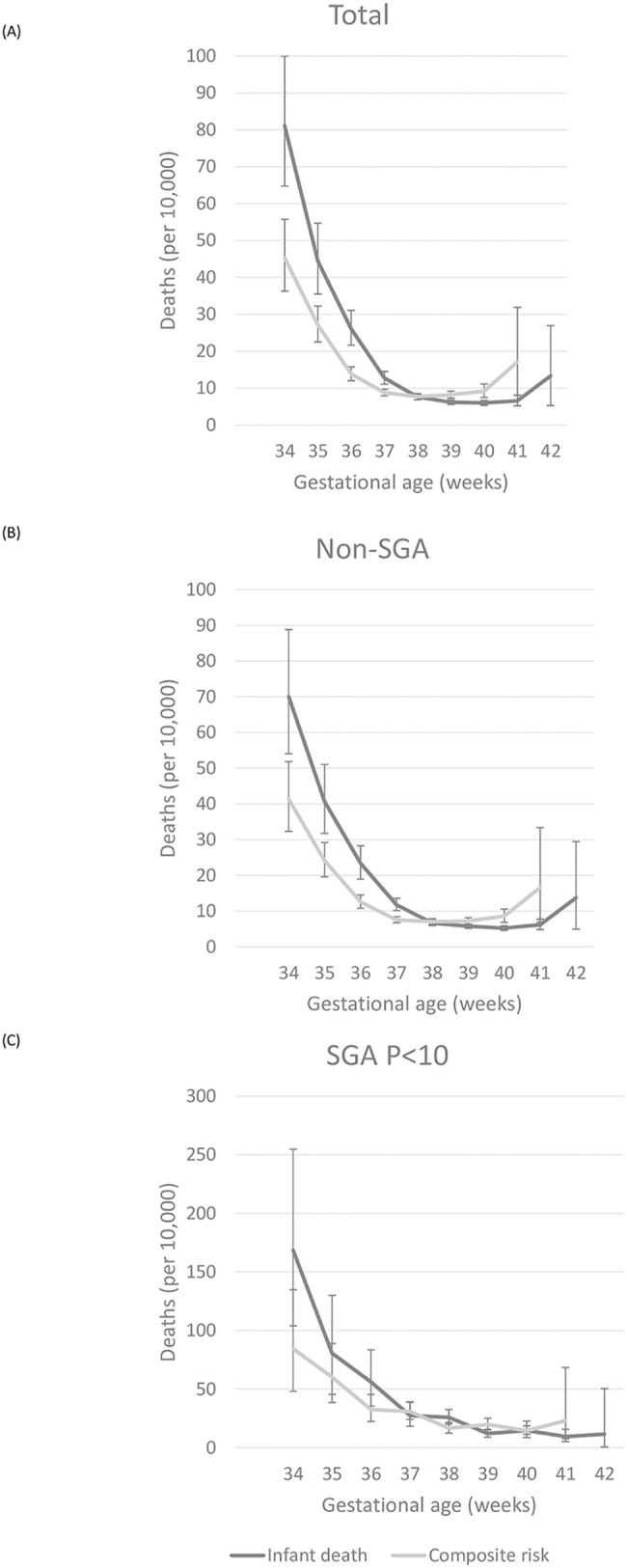
Graph comparing the risk of delivery (represented by infant death) with the risk of expectant management for 1 week (represented by the stillbirth rate plus the infant death risk at the subsequent gestational age) at each gestational age at late preterm and term. (A) Total population, (B) Non-small for gestational age (SGA) group, and (C) SGA group.

In the overall cohort, the highest risk of stillbirth was seen at 42 weeks with 8.84 per 10,000 ongoing pregnancies (95% CI 2.75–20.53 per 10,000) (Table 2 & Fig 2). Most stillbirths occurred of unknown causes (97.05%), followed by cardiopulmonary causes (0.1%) (Table 3). The highest risk of infant death was seen at 34 weeks with 81.11 per 10,000 live births (95% CI 64.75–99.98 per 10,000) (Table 2 & Fig 2). The risk of infant death at 37 weeks with 12.7 per 10,000 live births (95% CI 11.05–14.52 per 10,000) was 2.06 times higher than the risk at 39 weeks of gestation (6.17 per 10,000 live births, 95% CI 5.56–6.82 per 10,000). The most common etiology of infant death was cardiopulmonary 7causes, which accounted for 31.1% of all infant deaths among children born between 34 and 42 weeks, followed by sudden infant death syndrome (SIDS) (26.55%), accidents or injury (11.68%), neuromuscular causes (11.25%), and other causes (19.42%) (Table 3). At 37 weeks of gestation, the risk of expectant management was lower than the risk of delivery (8.25 compared with 11.82 per 10,000, relative risk 0.7, 95% CI 0.55–0.89) (Table 4). At 38 weeks of gestation, the risk of expectant management was similar with the risk of delivery: 7.6 (95% CI, 6.87–8.38) compared to 7.66 (95% CI, 6.87–8.5 per 10,000). The risk of expectant management became significantly higher than the risk of delivery at 39 weeks of gestation, and continued to diverge substantially at 40 and 41 weeks of gestation, favoring delivery over expectant management when considering the overall risk for either fetal or infant death (Fig 3). The absolute differences in risk, although statistically significant at 39 weeks of gestation and beyond, were small, ranging from 2.04 per 10,000 (95% CI 1.75–2.35 per 10,000) at 39 weeks of gestation to 10.58 per 10,000 at 41 weeks of gestation (95% CI 2.92–23.84 per 10,000). From these data, the number needed to deliver ranged from 4,914 (95% CI 4,262–5,711) at 39 weeks of gestation to 945 (95% CI 419–3,423) at 41 weeks of gestation (Table 4).

**Table 2:** Risk of stillbirth, infant death, and expectant management by gestational age.

Gestational Age (wk)	Stillbirth Total	Infant death Total	Deliveries	Stillbirth/10,000 Ongoing pregnancies (95%CI)	Infant death/10,000 Live births (95%CI)	Composite Risk of Expectant Management for 1wk*/10,000 (95%CI)
	**Total**					
**34**	179	81	10554	0.85(0.73,0.98)	81.11(64.75,99.98)	45.29(36.25,55.71)
**35**	202	82	19560	0.96(0.84,1.1)	44.39(35.48,54.67)	27.08(22.49,32.25)
**36**	217	116	47421	1.04(0.91,1.19)	26.06(21.6,31.08)	13.81(12.01,15.78)
**37**	228	206	174456	1.12(0.98,1.28)	12.7(11.05,14.52)	8.79(7.91,9.73)
**38**	261	390	547457	1.41(1.24,1.58)	7.6(6.87,8.38)	7.66(6.87,8.5)
**39**	277	367	628104	2.12(1.88,2.38)	6.17(5.56,6.82)	8.2(7.31,9.17)
**40**	172	309	544183	2.53(2.17,2.93)	5.97(5.33,6.67)	9.17(7.48,11.11)
**41**	49	81	129739	3.64(2.72,4.76)	6.51(5.2,8.03)	17.09(8.12,31.88)
**42**	4	6	4685	8.53(2.65,19.81)	13.29(5.29,26.92)	-
	**Non SGA**					
**34**	125	62	9407	0.64(0.53,0.75)	69.99(54.02,88.8)	41.31(32.31,51.85)
**35**	144	68	17806	0.74(0.62,0.86)	40.64(31.74,51.05)	24.08(19.59,29.21)
**36**	146	95	43656	0.75(0.64,0.88)	23.3(18.93,28.3)	12.56(10.79,14.5)
**37**	160	179	164567	0.84(0.72,0.98)	11.75(10.12,13.56)	7.53(6.7,8.42)
**38**	204	323	521425	1.18(1.03,1.35)	6.63(5.93,7.38)	6.98(6.21,7.82)
**39**	226	317	586392	1.87(1.64,2.13)	5.73(5.12,6.38)	7.17(6.3,8.11)
**40**	143	246	500970	2.3(1.95,2.7)	5.19(4.57,5.87)	8.57(6.85,10.57)
**41**	32	68	116033	2.67(1.85,3.7)	6.14(4.8,7.72)	16.52(6.86,33.36)
**42**	3	5	3812	7.86(1.96,20.38)	13.73(4.93,29.48)	-
	**SGA**					
**34**	54	19	1147	3.79(2.87,4.89)	168.44(103.94,254.81)	84.25(48.27,134.83)
**35**	58	14	1754	4.1(3.14,5.25)	80.46(45.41,129.94)	60.19(38.57,88.75)
**36**	71	21	3765	5.09(4,6.37)	56.09(35.43,83.5)	32.47(22.31,45.39)
**37**	68	27	9889	5.01(3.91,6.3)	27.38(18.31,39.02)	30.82(24.03,38.77)
**38**	57	67	26032	4.53(3.46,5.82)	25.8(20.11,32.47)	16.54(12.43,21.46)
**39**	51	50	41712	5.12(3.84,6.66)	12(8.98,15.64)	19.72(15.13,25.16)
**40**	29	63	43213	5.01(3.4,7.07)	14.6(11.29,18.5)	14.51(8.63,22.69)
**41**	17	13	13706	11.65(6.95,18.09)	9.49(5.22,15.62)	23.11(7.61,68.48)
**42**	1	1	873	11.44(0.65,50.28)	11.47(0.65,50.39)	-

CI, confidence interval.

*Composite risk = risk of stillbirth at this gestational age+risk of infant death at the next gestational age week., SGA, small for gestational age, wk, week

**Table 3:** Causes of stillbirth and infant death by gestational age, 34–42 weeks.

Death category	Stillbirth	SGA	Infant death	SGA
	Non-SGA		Non-SGA	
SIDS	·	·	372 (27.51)	60 (21.83)
Accident or injury	0	0	172 (12.72)	18 (6.55)
Related to labor and delivery, pregnancy complication	1 (0.08)	6 (1.48)	7 (0.52)	6 (2.18)
Infection	1 (0.08)	0	78 (5.77)	7 (2.54)
Cardiopulmonary	13 (1.10)	3 (0.74)	402 (29.73)	104 (37.81)
Hemorrhage	9 (0.76)	5 (1.23)	36 (2.66)	13 (4.72)
Gastrointestinal, endocrinology, Nephrology, Dermatology	7 (0.59)	1 (0.25)	57 (4.22)	18 (6.54)
Neuromuscular	0	0	157 (11.61)	26 (9.45)
Neoplasm	0	1 (0.25)	71 (5.25)	23 (8.36)
Unknown	1152 (97.38)	390 (96.06)	0	0
Total	1183	406	1352	275

SIDS, sudden infant death syndrome. SGA, small for gestational age, Data are n (%) or n.

**Table 4:** Relative and absolute risks of expectant management compared with delivery at 34–41 weeks of gestation.

	Total			Non-SGA			SGA		
Gestational age (wk)	Relative risk of expectant management compared with delivery	Absolute risk difference between expectant management and delivery	No. needed to deliver at this GA to prevent a single excess death	Relative risk of expectant management compared with delivery	Absolute risk difference between expectant management and delivery	No. needed to deliver at this GA to prevent a single excess death	Relative risk of expectant management compared with delivery	Absolute risk difference between expectant management and delivery	No. needed to deliver at this GA to prevent a single excess death
**34**	0.56(0.36, 0.86)	-35.82(-28.51, -44.27)	-	0.59(0.36, 0.96)	-28.67(-21.72, -36.95)	-	0.5(0.19, 1.3)	-84.19(-55.67, -119.98)	-
**35**	0.61(0.41, 0.91)	-17.31(-12.99, -22.42)	-	0.59(0.38, 0.92)	-16.55(-12.15, -21.83)	-	0.75(0.3, 1.95)	-20.27(-6.84, -41.19)	-
**36**	0.53(0.39, 0.73)	-12.25(-9.59, -15.31)	-	0.54(0.38, 0.77)	-10.75(-8.13, -13.8)	-	0.58(0.27, 1.28)	-23.62(-13.12, -38.11)	-
**37**	0.69(0.54, 0.88)	-3.92(-3.14, -4.79)	-	0.64(0.49, 0.83)	-4.23(-3.42, -5.14)	-	1.13(0.62, 2.12)	3.44(5.71, -0.24)	-
**38**	1.01(0.82, 1.24)	0.06(0, 0.12)	181818(86133, 5882353)	1.05(0.84, 1.32)	0.35(0.28, 0.44)	28490(22973, 36206)	0.64(0.38, 1.07)	-9.27(-7.68, -11.02)	-
**39**	1.33(1.07, 1.65)	2.04(1.75, 2.35)	4914(4262, 5711)	1.25(0.99, 1.58)	1.44(1.18, 1.73)	6958(5787, 8499)	1.64(0.97, 2.8)	7.72(6.15, 9.52)	1295(1050, 1625)
**40**	1.54(1.12, 2.08)	3.2(2.14, 4.45)	3128(2249, 4665)	1.65(1.17, 2.31)	3.38(2.28, 4.7)	2957(2126, 4385)	0.99(0.47, 2.01)	-0.09(-2.66, 4.19)	-
**41**	2.62(1.01, 6.14)	10.58(2.92, 23.84)	945(419, 3423)	2.69(0.89, 6.96)	10.38(2.06, 25.64)	963(390, 4843)	2.43(0.49, 13.11)	13.62(2.38, 52.86)	734(189, 4197)
**42**	-	-	-	-	-	-	-	-	-

CI, confidence interval., SGA, small for gestational age, wk, weeks, GA, gestational age, No, number

### Non-SGA group

In the non-SGA group, the risk patterns of stillbirth, infant death, and composite death were similar to those seen in the total group, according to GA (Table 2, Figs 2 & 3). The risk of infant death at 38 weeks (6.63 per 10,000, 95% CI 5.93–7.38) was not significantly greater than that at 39 weeks (5.73 per 10,000, 95% CI 5.12–6.38). At 38, 39 and 41 weeks of gestation, the risk of expectant management was higher than the risk of delivery, although the CIs overlapped (Table 2). The risk of expectant management was significantly higher than the risk of delivery at 40 weeks of gestation, the risk of expectant management was 8.57 per 10,000, whereas the risk of delivery was 5.19 per 10,000 (relative risk 1.65, 95% CI 1.17–2.31) (Table 4). The absolute risk differences, although statistically significant at 39 weeks of gestation and beyond, are small, ranging from 1.44 per 10,000 (95% CI 1.18–1.73 per 10,000) at 39 weeks of gestation to 10.38 per 10,000 at 41 weeks of gestation (95% CI 2.06–25.64 per 10,000). The number needed to deliver ranged from 6,958 (95% CI 5,787–8,499) at 39 weeks of gestation to 963 (95% CI 390–4,843) at 41 weeks of gestation.

### SGA group

In SGA pregnancies, the risk of stillbirth was significantly greater at all GAs, except 42 weeks, than it was for non-SGA pregnancies (Table 2 & Fig 2). The risk of stillbirth increased abruptly, at 41 weeks with 11.65 per 10,000 ongoing pregnancies (95% CI 6.95–18.09), compared to 39 weeks with 5.12 per 10,000 ongoing pregnancies (95% CI 3.84–6.66). The risk of infant death at 38 weeks with 25.8 per 10,000 live births (95% CI 20.11–32.47 per 10,000), which were significantly higher than the risk at 39 weeks of gestation (12 per 10,000 live births, 95% CI 8.98–15.64 per 10,000). There was no significant difference between the risk of expectant management and the risk of delivery, at any GA (Table 2 & Fig 3). An absolute difference in risk was seen at 39 weeks of gestation, 7.72 per 10,000 (95% CI 6.16–9.52 per 10,000), vs. 41 weeks of gestation, 13.62 per 10,000 (95% CI 2.38–52.86 per 10,000) (Table 4). The number needed to deliver was 1,295 (95% CI 1,050–1,625) at 39 weeks of gestation and 734 (95% CI 189–4,197) at 41 weeks of gestation.

## Discussion

In this study, we estimated the composite risk of stillbirth plus infant death to represent the risk of expectant management for each 1 additional week of pregnancy. In Korean women, the risk of expectant management was lower in late preterm pregnancies and at 37 weeks of gestation than the risk of delivery, became equal at 38 weeks of gestation, and then exceeded the risk of delivery at 39, 40, and 41 weeks of gestation. This result was similar to those of previous studies done in the USA, including on individuals of white, black, Latina, and Asian descent [5, 14]. However, in the previous study, the risk of infant death in Asians abruptly increased at 41 weeks and beyond, which was similar to the risk pattern of infant death in the Hispanic population [5]. In white and black individuals, the risk of infant death significantly increased after 41 weeks. Research on migrant health has shown that immigrant status affects maternal and infant health outcomes, because mothers and infants comprise a vulnerable segment of society that faces health challenges that differ from those of other populations [15–18]. To avoid this bias, we excluded births from women of non-Korean nationalities. Although we could not exclude pregnancies complicated by diabetes mellitus and chronic hypertension, owing to limitations in the national dataset and a lack of availability of medical records specific to mother–infant pairs, the current study demonstrated that the risk patterns of stillbirths and infant deaths were stratified by gestational age in Korean women in a similar manner as the risk patterns of white women in a previous study [5]. The infant mortality rates in late preterm and early term births, stratified by gestational week and birth weight, in Asian countries, have not been addressed, before. The risk of infant death during late preterm in Korean women in the current study seemed substantially higher than it in the previous study of USA [19]. It is well known that maternal risk factors and obstetric complications are significantly higher in late preterm births than in term births and women with medically indicated late preterm birth are older and mainly nulliparous [7, 8, 20]. We speculate that high risk of infant death in Korean during late term might be related with increasing tendency of old aged nulliparous women and low birth rate in Korea, as well as not excluding complicated pregnancies. In addition, the risk of stillbirth per 10,000 ongoing pregnancies and infant death per 10,000 live births during term were quite a bit lower in the current study compared to in Asian populations in the previous study [5], even though we included complicated pregnancies which would be expected to negatively affect fetal/infant health outcomes. Racial disparity about the intrauterine maturation or the other factors needs to be further studied.

In terms of early term birth, there was no significant difference in the risk of infant death, between 38 and 39 weeks of gestation, in the non-SGA group. Recent studies suggested that neonatal morbidities between 38 weeks 39 weeks are smaller than previously anticipated [21, 22]. Although we cannot fully apply this to women who are scheduling elective cesarean delivery, it may be acceptable to schedule elective cesarean delivery a few days prior to 39 weeks 0 days, in a fetus of appropriate weight for gestational age.

The current study has some of the same methodologic limitations as previous studies [5, 14]. Another limitation is our inability to exclude some complicated pregnancies owing to limitations in the data. The other potential limitation of the study is that stillbirths may have occurred one or more days before actual delivery due to delayed diagnosis or delivery. These delays would lead to the authors’ findings being biased towards lower rates of fetal death at each gestational age, since the stillbirths would have been recorded at a later gestational age. However, one of the strengths of the current study is that the dataset is large enough to examine stillbirth rates at each gestational age in pregnant Korean women. To exclude bias from maternal race and the socioeconomic differences experienced by immigrant women in Korea, women with other nationalities aside from Korean were excluded, although it made that the study results are only valid for Korean population. Another strength of this study is that we could compare our data with data from previous studies [5,14] and prove that the risk of infant death does not increase at 41 weeks, but increase after 41 weeks, at least in Korean women, even including complicated pregnancies. To decrease a risk of infant death in the births after 41 weeks, study results support an induction of delivery before 42 weeks. Lastly, the current study divided the population according to birth weight. In the SGA population, the risk of infant death was significantly increased until 38 weeks, and the risk of stillbirth was significantly increased after 40 weeks compared to 39 weeks, within the SGA population. Another US national cohort study also showed a similar stillbirth pattern in SGA pregnancies in less than the 10th percentile of birth weight, but did not include infant death data [23]. A previous randomized trial about Induction versus expectant monitoring for intrauterine growth restriction at term showed no significant differences in adverse outcomes between induction of labor and expectant monitoring until 40 weeks’ gestation [24]. Therefore, our results are similar with the results of the DIGITAT trial [24], but our results support delivery before 41 weeks when we suspect fetal growth restriction less than 10 percentile, to decrease the risk of stillbirth. Although ACOG guideline suggests a delivery at 38 0/7–39 6/7 weeks of gestation in cases of isolated fetal growth restriction, our study results suggest that physicians should be careful about a delivery before 39 weeks, without medical indication, in SGA pregnancies. Because our current cohort study relies on retrospective cohort data and does not consider maternal and neonatal short-term and long-term morbidity, further research should be directed to evaluate the optimal time of delivery in SGA fetuses. Meanwhile, the optimal time of delivery for uncomplicated SGA in Korean pregnant women can be suggested as between 39 and 40 weeks of gestation to decrease risks of infant deaths and stillbirths.

## Conclusion

In pregnant Korean women, a delivery at 39 0/7–41 6/7 weeks of gestation can minimize the risks of stillbirth and infant death. However, it may be acceptable to schedule elective cesarean delivery a few days prior to 39 weeks 0 days when the estimated fetal body weight is within the normal range. Decisions regarding timing of delivery of SGA fetuses must be individualized. However, if there is no medical indication, delivery between 39 and 40 weeks can be considered to decrease risk of infant death and stillbirths, in uncomplicated SGA pregnancies. Once again, further study including maternal and infant morbidity rates are still needed to evaluate the optimal time of delivery, in consideration of maternal race, age, fetal growth, and so on.
